# Detection of Superior Vena Cava Obstruction on Dynamic 99mTc-DTPA Renal Transplant Scintigraphy

**DOI:** 10.4274/mirt.02986

**Published:** 2016-02-10

**Authors:** Elahe Pirayesh, Hamidreza Hashemifard, Majid Assadi

**Affiliations:** 1 Shahid Beheshti University of Medical Sciences, Shohada-e-tajrish Medical Center, Department of Nuclear Medicine, Tehran, Iran; 2 Sabzevar University of Medical Sciences, Vasie Hospital, Department of Radiation Oncology, Sabzevar, Iran; 3 Bushehr University of Medical Sciences, The Persian Gulf Nuclear Medicine Research Center, Bushehr, Iran

**Keywords:** Superior vena cava obstruction, Radionuclide imaging, kidney

## Abstract

We present an asymptomatic patient with a history of prolonged hemodialysis through a right internal jugular vein catheter who was diagnosed with superior vena cava (SVC) obstruction on 99mTechnetium-diethylenetriaminepentaacetic acid renal transplant scintigraphy. During the angiographic phase, an unusual vascular filling pattern was detected on the anterior view of the abdomen. Angioscintigraphic imaging of the chest wall was suggestive of SVC obstruction. The SVC obstruction in our patient was related to the long-term use of an indwelling catheter in the central venous system, which is a well-known complication of such a procedure. There is also evidence of a hypercoagulable state in dialyzed uremic cases; therefore, our patient may have been more susceptible to an SVC thrombosis. Acquired compensatory dilatation of the azygos vein is rather a rare finding. To the best of our knowledge, this is the first report describing an asymptomatic patient with SVC obstruction who was diagnosed by renal scintigraphy.

## Abstract (tr)

Bu yazıda, uzun süre sağ internal juguler venöz kateter yolu ile hemodiyaliz geçmişi olan ve yapılan 99mTc-DTPA renal transplant sintigrafisinde superior vena kava (SVK) obstrüksiyonu saptanan asemptomatik bir hasta sunulmaktadır. Anjiografik fazda batının önden görüntülenmesi sırasında sıradışı bir vasküler doluş paterni saptandı. Toraks anjiosintigrafik görüntülemesi bulguları SVK obstrüksiyonu ile uyumlu idi. Bizim hastamızda SVK obstrüksiyonu, bu işlemin iyi bilinen bir komplikasyonu olan, santral venöz sistemde uzun süreli tutulan katetere bağlı idi. Diyaliz uygulanan üremik hastalarda hiperkoagülabilite oluştuğuna dair kanıt bulunmaktadır, bu bizim hastamızın SVK trombozuna yatkın olmasına neden olmuş olabilir. Azigos veninin edinsel kompansatuvar dilatasyonu oldukça nadir bir bulgudur. Bu yazı bilgimiz dahilinde renal sintigrafi ile SVK obstrüksiyonu tanısı konulan asemptomatik bir hastanın bildirildiği ilk yayındır.

## INTRODUCTION

Superior vena cava (SVC) obstruction is usually secondary to extraluminal compression of the vein. However, intraluminal obstruction secondary to a tumoral infiltration or the more common thrombosis are increasing and they currently account for at least 40% of all cases. This increasing rate is associated with the increasing use of indwelling central venous devices, which render the SVC susceptible to thrombosis. The development of clinical signs and symptoms depends on the severity and rapidity of SVC obstruction (1,2).

## CASE REPORT

**Figure 1:**
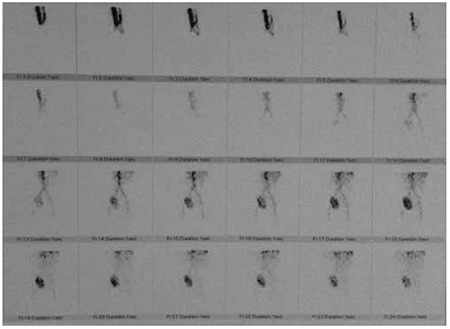
Dynamic renal scintigraphy, anterior view after bolus injection of 7 mCi 99mTechnetium-diethylenetriaminepentaacetic acid through an 18-gauge venous cannula in the right antecubital vein. In the angiographic phase, sequential 1-second images revealed an unusual vascular filling pattern along with patent iliac arteries and the transplanted kidney

**Figure 2:**
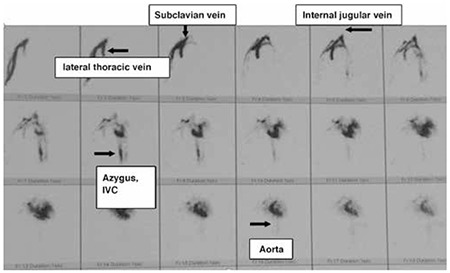
Angioscintigraphy anterior view of the chest after bolus injection of 5 mCi 99mTechnetium-diethylenetriaminepentaacetic acid through the same venous cannula. Sequential 1-second images showed rapid filling of the right basilic and cephalic veins, followed by axillary and subclavian veins. Two parallel channels appeared and then the right heart, left heart and aorta became visible in turn. Regurgitation into the lateral thoracic and internal jugular veins was also noted IVC: Inferior vena cava

A 21-year-old female, who has underwent renal transplantation 1 week ago, was referred to the department of nuclear medicine for renal transplant scintigraphy. She had end stage renal disease as a consequence of nephritic syndrome for the past 4 years, and was maintained on regular hemodialysis. Both her previous right and left brachial-cephalic arterio-venous fistulas had become dysfunctional, and she had been on a renal transplant waiting list since then. A catheter had been implanted in her right internal jugular vein (IJV), and she underwent hemodialysis via this route for 10 months. The catheter has been removed a few days after transplantation. Dynamic renal scintigraphy was obtained in an anterior view after bolus injection of 7 mCi 99mTechnetium-diethylenetriaminepentaacetic acid (^99m^Tc-DTPA) through an 18-gauge venous cannula in the right antecubital vein intravenously. The images were obtained with a single-head gamma camera (Siemens ecam) fitted with a low energy high-resolution collimator. In the angiographic phase, sequential 1-second images revealed an unusual vascular filling pattern along with patent iliac arteries and transplanted kidney (Figure 1). In order to gather further information on the abnormal finding, an additional angioscintigraphy of the chest in the anterior view was performed following bolus injection of 5 mCi ^99m^Tc-DTPA through the same venous cannula 4 hours later (Figure 2). Sequential 1-second images demonstrated rapid filling of the right basilic and cephalic veins followed by the axillary and subclavian veins (Figure 2). Unexpectedly, two parallel channels appeared and the right heart, left heart and aorta became visible in turn. Regurgitation into the lateral thoracic and IJVs was also noted (Figure 2). All findings were suggestive of SVC obstruction, which was attributed to the prolonged IJV catheterization, accompanied by blood flow through the azygos system (Figure 2). The patient did not have any symptoms or signs, except some dilated collateral veins over the chest and abdominal wall that were retrospectively detected on physical examination. Due to the risk of contrast nephropathy and the patient’s being asymptomatic, the referring physician did not agree to perform computed tomography (CT) angiography to obtain detailed anatomical information.

## DISCUSSION

Thrombosis is a relatively common complication in patients with central venous catheterization, detected in up to 40% of such patients. Additionally, SVC obstruction is a frequent complication of IJV catheterization. Most of these patients remain asymptomatic since the thrombosis develops gradually permitting the time for development of collateral drainage making the symptoms less prominent or even undetectable (1,3). In addition to these, SVC thrombosis results in impaired venous drainage and increased flow in the azygos and hemiazygos systems, which divert blood from the SVC into the inferior vena cava. Therefore, compensatory dilatation and tortuosity of the azygos veins may be detected. Depending on the level of the SVC occlusion relative to the azygos arch, different patterns of collateral vessels and bypass routes may be viewed. The most common veins that are involved are the internal thoracic, lateral thoracic and intercostal veins (4). In a study by Podoloff and Kim (5), upper extremity radionuclide venograms were performed on 220 patients. An evidence of obstruction, collateral flow without an obstruction and a slow-flow pattern were observed in 123, 6 and 12 patients respectively. The upper extremity contrast venography performed within 48 hours of the radionuclide venogram, yielded the same result in 19 of the 26 patients (16 correctly diagnosed as obstructed, three correctly diagnosed as unobstructed). Six patients had the slow-flow pattern without collaterals or obstruction. Subsequent follow-up contrast studies of these six patients revealed no evidence of obstruction or collaterals. The authors concluded that obstruction with collateral flow on radionuclide venograms correctly predicted obstruction. In another study, a male patient with lung cancer underwent Tc-99m red blood cell gastrointestinal bleeding study due to bloody stool. In the first-pass phase, there was early filling of the radiotracer to the inferior cava along with an abdominal aortic aneurysm. Collateral circulation was detected in the trunk on the subsequent blood-pool images. A successive radionuclide SVC study confirmed SVC obstruction just above its entrance to the right atrium. A similar issue was confirmed in our patient by the chest CT scan with i.v. contrast (6). A Chest CT scan with i.v. contrast is the preferred diagnostic method to confirm the presence of SVC obstruction and collateral channels (1). However; because of the inherent risk of contrast injection especially in a transplanted kidney (7) and in view of the fact that anatomical details obtained by CT scan are not always required for clinical management, radionuclide angiography is accepted as an accurate, noninvasive and convenient procedure with a lower radiation dose that can be used for the diagnosis and treatment monitoring of these patients (8). The SVC obstruction in our patient was related to the long-term use of an indwelling catheter in the central venous system, which is a well-known complication of this procedure. There is also evidence of a hypercoagulable state in uremic patients undergoing dialysis (9), which may have caused our patient to be more susceptible to an SVC thrombosis. Acquired compensatory dilatation of the azygos vein is rather a rare finding (10). Besides, to the best of our knowledge, this is the first report describing an asymptomatic patient with SVC obstruction diagnosed on renal scintigraphy.
